# Animal Female Meiosis: The Challenges of Eliminating Centrosomes

**DOI:** 10.3390/cells7070073

**Published:** 2018-07-10

**Authors:** Oliver J. Gruss

**Affiliations:** Institute of Genetics, University of Bonn, 53115 Bonn, Germany; ogruss@uni-bonn.de; Tel.: +49-0228-73-4258; Fax: +49-0228-73-4263

**Keywords:** meiosis, oocyte, centrosome elimination, spindle self-assembly, RanGTP, centrosome reassembly

## Abstract

Sexual reproduction requires the generation of gametes, which are highly specialised for fertilisation. Female reproductive cells, oocytes, grow up to large sizes when they accumulate energy stocks and store proteins as well as mRNAs to enable rapid cell divisions after fertilisation. At the same time, metazoan oocytes eliminate their centrosomes, i.e., major microtubule-organizing centres (MTOCs), during or right after the long growth phases. Centrosome elimination poses two key questions: first, how can the centrosome be re-established after fertilisation? In general, metazoan oocytes exploit sperm components, i.e., the basal body of the sperm flagellum, as a platform to reinitiate centrosome production. Second, how do most metazoan oocytes manage to build up meiotic spindles without centrosomes? Oocytes have evolved mechanisms to assemble bipolar spindles solely around their chromosomes without the guidance of pre-formed MTOCs. Female animal meiosis involves microtubule nucleation and organisation into bipolar microtubule arrays in regulated self-assembly under the control of the Ran system and nuclear transport receptors. This review summarises our current understanding of the molecular mechanism underlying self-assembly of meiotic spindles, its spatio-temporal regulation, and the key players governing this process in animal oocytes.

## 1. Defining the Biological Problem of Centrosome Elimination in Animal Oocytes

**Figure 1:**
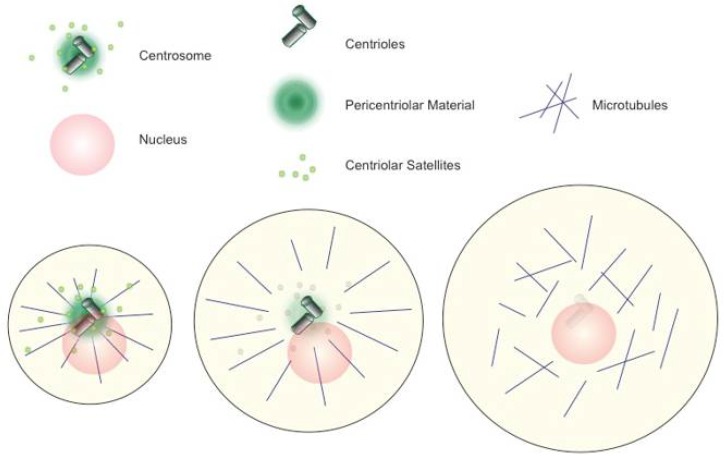
Loss of centrosomes during metazoan oogenesis. During their long growth phase, metazoan oocytes eliminate centrosomes. Elimination starts from the pericentriolar material (PCM) and ends with depletion of centrioles, mostly before entry into meiosis.

Animal reproductive cells have not only evolved ways to reduce their chromosome content but also have acquired specialised cell morphologies to cope with the diverse cellular functions required for fertilisation. Animal oocytes generally grow up to very large sizes. They accumulate energy stocks and store mRNAs and proteins, which will enable rapid cell divisions without the need for the duplication of cellular contents right after fertilisation (“cleavage divisions”). Although microtubule functions are required for oocyte development and maturation, almost all metazoan oocytes eliminate their centrosomes, i.e., major microtubule-organizing centres (MTOCs), during or right after the long growth phases of oogenesis [1,2,3], Figure 1.

Generally, an animal centrosome comprises a pair of centrioles embedded into pericentriolar material (PCM). Centrioles consist of barrel-shaped microtubule bundles of defined length arranged with nine-fold symmetry, usually in microtubule triplets [4], around which the PCM assembles. The PCM was originally referred to as an “amorphous mass of proteins” but proved highly organised. It consists of concentric functional layers, which cluster proteins of particular functions [5]. The γ-tubulin ring complex (γ-TuRC), for instance, concentrates in one of the PCM-layers that wrap around centrioles [6,7]. Proteins or protein modules such as the γ-TuRC accumulate in the PCM in high local concentration building a “selective” phase designed for microtubule assembly and organisation, as well as other centrosome-associated functions [8,9,10]. The PCM-localised nucleation and minus end capping activities of γ-TuRCs explain the typical microtubule array radiating out from the centrosome in animal cells. During the cell cycle, the pair of centrioles (referred to as mother centrioles) splits to allow the assembly of two new daughter centrioles, separately assembling on each of the mothers. When a cell enters mitosis, the two pairs of centrioles build up two independent mitotic PCM structures. These quickly permit the growth of two separating centres of mitotic microtubule arrays in a balanced fashion to initiate spindle formation and to maintain robust spindle bipolarity [11,12,13]. Systematic knockdown approaches in cell culture along with proteomic studies and super-resolution microscopy have pushed forward our molecular understanding of structure, function and replication capacity of centrosomes in the last decades. Different aspects of centrosome biology have been comprehensively overviewed in a number of excellent reviews, to which I refer for further details (these include but are not limited to: [14,15,16,17,18,19,20]). PCM and centrioles are surrounded by small proteinaceous structures termed centriolar satellites. Analysing the protein composition and the functions associated with them has brought centriolar satellites back into the focus of centrosome research ([21,22,23], most recently discussed in Tollenaere et al., in this issue of Cells). Many defining proteins of centrioles, PCM or satellites are exclusive to these individual centrosome compartments, while other proteins are shared between the compartments. Centrosomal proteins, alone or regularly in high molecular weight complexes, collectively carry the overall function of centrosomes, in particular in nucleating and organising microtubules, as well as during the single round of centrosome replication before cell division.

Evidence for centrosome elimination from animal oocytes initially came from electron microscopy (EM) analysis of oocytes from different species. Here, centrioles are readily visible only during early or intermediate stages of oogenesis [24,25,26,27,28]. Blocking centrosome loss in different metazoan model systems revealed its physiological importance: oocytes ought to eliminate centrosomes in order to avoid a superior number after fertilisation, which would otherwise result in multipolar spindle formation and chromosome missegregation [29,30,31,32]. Elegant studies in sea urchins and flies documented details about timing and molecular determinants of centrosome elimination in metazoan oocytes. Starfish oocytes abolish centrosomes late during the asymmetric cell divisions of meiosis I and II. The starfish uses the two consecutive meiotic divisions not only to dispose of the surplus chromosomes when gaining a haploid genome, but also dumps 3 out of 4 centrioles into the polar bodies, which divide from the large oocyte. The last daughter centriole gets degraded soon after meiosis II in the oocyte cytoplasm. These observations support the idea of an active mechanism to eliminate centrioles in oocytes [33]. Studies in *Drosophila* provided further evidence for this hypothesis. Here, centriole elimination occurs during oogenesis long before meiosis, like in most metazoan oocytes. It requires the inactivation of Polo1 kinase, proceeds by removing PCM proteins, and ends with the complete loss of all centrioles [29,34], Figure 1.

**Figure 2:**
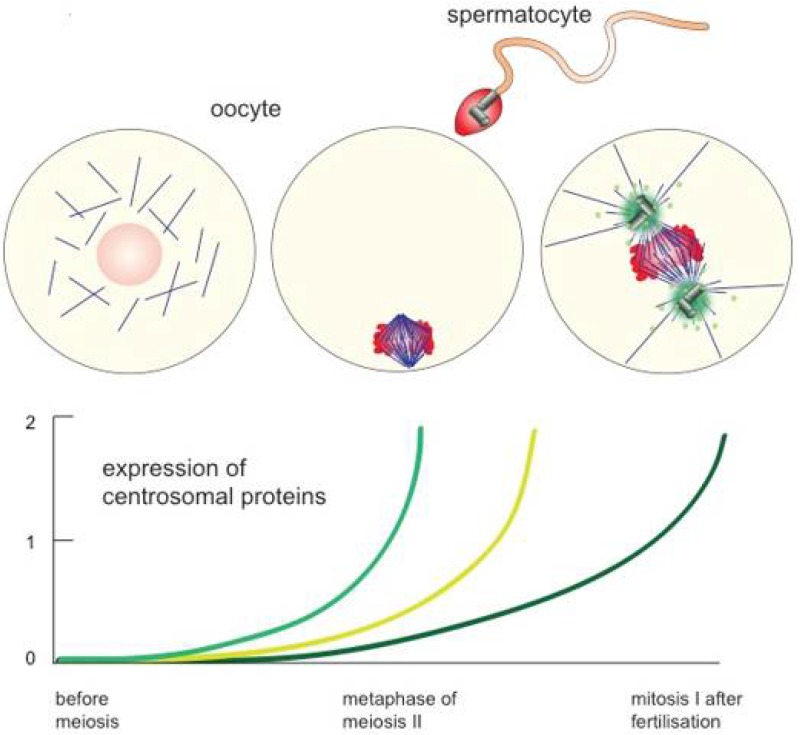
Re-assembly of zygotic centrosomes. Metazoan oocytes lose their centrosomes regularly before meiosis but rebuild them only after fertilisation. Oocytes use the sperm-derived basal body as a platform for the first centrosome assembly and restart the expression of centrosomal proteins (see coloured lines in graph) to allow on-going centrosome replication afterwards. Female meiosis, however, occurs in most metazoan species in the absence of centrosomes.

Centrosome elimination during oogenesis seems to be incompatible with the need for microtubule organisation during female meiotic spindle formation as well as pronuclear migration upon zygote formation. Moreover, mitotic spindle formation and spindle positioning during the cell divisions of early embryogenesis necessitate interactions between the spindle and the cell cortex. The defined orientation of the spindle is a structural basis for cell differentiation in early embryos. Hence, centrosomes need to establish interactions of the spindle with the cortex and the plasma membrane via their cytoplasmic (“astral”) microtubules [35,36,37]. Additionally, spindle formation ought to be extremely fast during the short cell cycles in early embryos. Fast spindle assembly kinetics requires active MTOCs [38]. The centrosome is, therefore, generally re-established upon fertilisation, or right afterwards during early embryogenesis. In most animal species, sperm components (i.e., the basal body or respective remnants) trigger the reassembly of centrosomes in the cytoplasm of the zygote [39], Figure 2.

In general, animal sperm are characterized by extremely compacted chromatin and reduced cytoplasmic volume as well as an MTOC, which is often stripped down to a single basal body. The basal body organises the microtubules of the flagellum that drives sperm motion. Although the sperm basal body and its associated structure (daughter centriole or often only a centriole remnant) generally define the platform for zygotic centrosome reformation, the process significantly differs from centrosome duplication in somatic cells, as sperm centrioles undergo various modifications and structural changes that seem to limit their duplication capacity [40]. The sperm basal body and the daughter centriole remnant still work as platforms for the first round of centriole duplication in the cytoplasm of the fertilised ovum. The particular morphology of sperm centrioles and the physiological consequences of their morphogenesis are reviewed in this issue of Cells by Tomer Avidor-Reiss and colleagues.

## 2. Female Meiotic Spindle Formation: Self Assembly of Bipolar Structures

**Figure 3:**
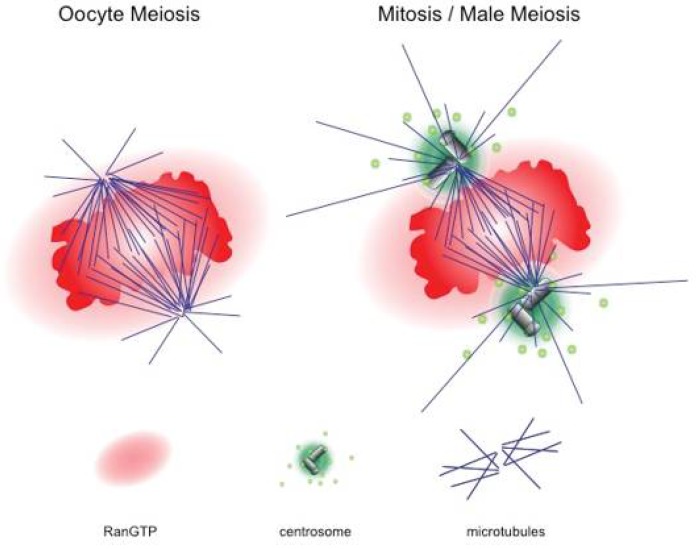
Oocyte meiosis versus mitosis. While spindle formation occurs in the absence of centrosomes in female animal meiosis, mitosis in early cell divisions after fertilisation, mitosis in somatic cells and male meiosis ensues in the presence of two centrosomes as microtubule-organizing centres (MTOCs). In both situations, a high concentration of RanGTP surrounds the chromatin.

As microtubule organisation remains important for oocyte meiosis as well as for pronuclear migration upon fertilisation, centrosome elimination before female meiosis leaves a gap, in which microtubule nucleation and organisation occur in the absence of the canonical animal MTOCs, Figure 2. Oocytes of most animal species go through the two meiotic divisions without centrosomes, i.e., preformed MTOCs, Figure 2 and Figure 3.

The progression of oocytes through the meiotic divisions often takes several hours. Compared to the rapid mitotic phases after fertilisation, which regularly run in less than 60 min, female meiosis is slow [38] and more frequently results in chromosome segregation errors than in somatic cells, in particular in mammals [41]. In fact, 80% of human oocytes divide faithfully, but up to 20% show aneuploidy, arising both from defects in meiotic recombination and chromosome segregation errors [42,43,44,45]. Going through meiosis II normally involves an arrest in metaphase, in which the ovum awaits fertilisation. The centrosome-free bipolar structure of the meiosis II spindle stays with aligned chromosomes and stable microtubule-to-kinetochore attachment for many hours, despite the dynamic behaviour of spindle microtubules. These observations pose the question of how microtubules can assemble stable meiotic spindles without a predefined starting point. In mitosis, the pair of centrosomes mark the two poles of mitotic spindles and define the axis, along which chromosome segregation takes place. Here, the microtubule nucleating and organising activities densely packed in the PCM of the centrosome pair will readily start the assembly of microtubules at two defined start points (Figure 3). Microtubule assembly in the absence of centrosomes requires an alternative trigger for nucleation and organisation. Experiments in amphibian oocytes initially showed that the chromatin takes on the active role in spindle formation [46,47], Figure 3. Of note, chromatin does not provide a platform for spindle formation like centrosomes but rather “instructs” the neighbouring cytoplasm to start microtubule formation [48]. Microtubule nucleation around chromatin proceeds slowly compared to centrosomal nucleation. However, chromatin-induced microtubule assembly results in proper bipolar spindle formation with time [49]. The main principles and molecular details of centrosome free spindle formation were worked out using cell-free extracts from amphibian eggs, in particular those of *Xenopus laevis*, as well as intact metazoan oocytes.

Cell-free extracts of *Xenopus* eggs preserve the natural arrest in metaphase II of meiosis [50]. The formally meiotic egg extracts recapitulate general principles of microtubule assembly seen both during meiotic spindle formation in oocytes, and mitotic spindle assembly in embyros and somatic cells. When isolated nuclei, regularly associated with a centrosome, come into the reaction, these will follow the cell cycle, recapitulate centrosome and chromosome duplication at low Cyclin-dependent kinase (Cdk) 1 activity, and build up bipolar spindles when the Cdk1 activity rises [51,52]. Inducing the cycling reaction in frog extracts in the presence of sperm results in chromatin decondensation, its duplication, re-condensation and, finally, its alignment on the metaphase plate of a bipolar spindle. The female cytosol turns back the basal body of the sperm and its daughter centriole into functional centrosomes. The first centrosome pair duplicates concomitant with chromosome replication, yielding two centrosomes defining the two poles of the spindle. Remarkably, however, these extracts also promote spindle formation around chromatin without centrosomes, as an oocyte would do in meiosis. Even the addition of artificial chromatin will lead to the formation of bipolar microtubule arrays that resemble centrosome-free meiotic spindles of intact oocytes concerning size and organisation [53,54]. Although oocytes and eggs represent very specialised cell types, the molecular analysis of chromatin-driven, meiotic spindle formation in egg extracts strongly influenced our view of the principles of spindle formation in meiosis and mitosis (collectively being referred to here as M-phase).

## 3. The Role of RanGTP in Meiotic Spindle Formation

**Figure 4:**
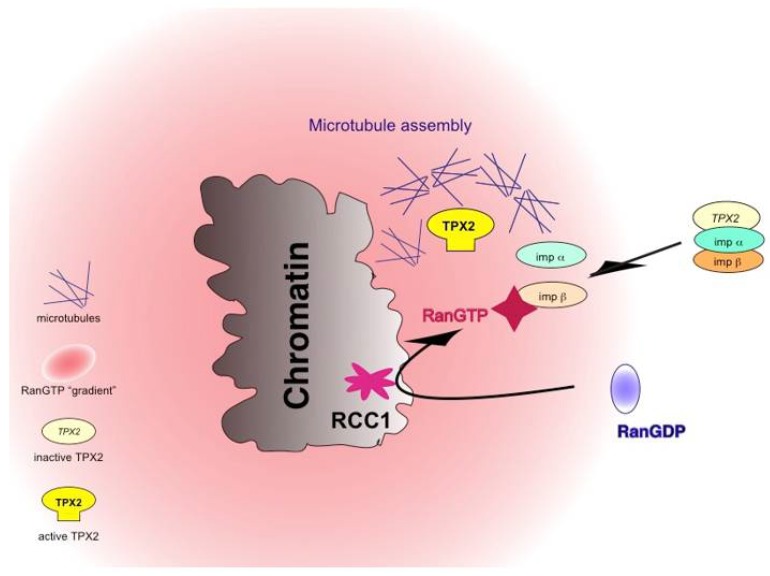
Paradigm of the mechanism of Ran in spindle formation. RanGTP activates numerous spindle assembly factors by release from nuclear import receptors (imp, importin, shown here) or, alternatively, association with nuclear export receptor CRM1 (not shown, see text). Please note that importin can be only importin β, or the importin β/importin7, or the importin α/β heterodimer. The production of RanGTP depends on the activity of the chromatin protein Rcc1, the G-nucleotide exchange factor of Ran.

Using these egg extracts, it could be shown that RanGTP plays a key role for chromatin driven processes in spindle formation. As a member of the Ras-superfamiliy of small GTPases, Ran’s nucleotide cycle relies on several accessory factors. The highly conserved chromatin-bound protein RCC1 (Regulator of Chromosome Condensation) is the only known G-nucleotide exchange factor for Ran and converts RanGDP to RanGTP on chromatin (Figure 4).

The active, GTP-bound form of Ran requires Ran binding proteins 1 or 2 (RanBP1/2) and the Ran GTPase activating protein (RanGAP) for GTP hydrolysis [55]. Directly adding recombinant Ran and its associated proteins to *Xenopus* egg extracts allows manipulating endogenous RanGTP levels. Blocking RanGTP production completely abolishes spindle formation around artificial chromosomes [56], while it has no influence on microtubule nucleation from isolated centrosomes [56]. The “opposite” experiment, i.e., increasing RanGTP levels, stimulates the nucleation of a radial microtubule array (“aster”) formed around both isolated centrosomes [57] and sperm centrioles that stay in the vicinity of chromatin [58,59,60,61]. The addition of recombinant RanGTP to *Xenopus* cell-free extracts influences dynamics of microtubules nucleated from centrosomes [60,61,62] but also leads to the formation of spindle-like structures in the absence of chromatin or centrosomes [56,58,60,63,64]. The activity of RanGTP in inducing the formation of microtubule assemblies in M-phase independently of any particular source shows that RanGTP, directly or indirectly, mediates a variety of different processes in spindle formation during the M-phase. The analogy to Ran’s main function in interphase nuclei has helped to explain how Ran functions in mitosis. Nuclear import works via a family of related nuclear import receptors (importins), which bind nuclear proteins in the cytoplasm and promote their entry into the nucleus. Inside the nucleoplasm, RanGTP directly binds to these importins, induces a conformational switch, and liberates the bound nuclear protein inside the nucleus. In turn, nuclear RanGTP can pick up a protein destined for nuclear export together with an export receptor (exportin) to mediate protein export. Indeed, it could be demonstrated that RanGTP activates numerous microtubule regulators using the same conformational switches in M-phase on two nuclear transport receptors—importin β and exportin1/CRM1—to locally activate (importin β), or to specifically target (exportin 1) downstream activities in spindle formation. Among these mechanisms, the release from importin β is the predominant mechanism of target activation in the M-phase (Figure 4). Proteins can interact with importin β directly or via adaptor proteins, such as importin α. In either case, RanGTP binds to importin β and releases the bound factors upon inducing a conformational switch in importin β. Most factors identified as Ran targets in the M-phase show direct importin α binding via a nuclear localisation signal (NLS). The microtubule associated proteins TPX2 (targeting protein for XKlp2) [65] and NuMA (nuclear protein of the mitotic apparatus) [66,67], for instance, are activated by RanGTP when released from importins and localize to the spindle in M-phase in a Ran-GTP dependent manner. In interphase, when the nucleus is intact, the NLS confers importin binding in the same manner. Here, importin mediates nuclear import, and RanGTP releases the nuclear protein from importin in the nucleus [68]. The interaction of TPX2 with importin α, therefore, has two consequences: first, it mediates nuclear import of the protein in interphase and, second, it regulates its activity in mitosis. The interaction is special as the key NLS in TPX2 contacts the minor binding site in importin α, while most other NLS proteins interact with importin α’s major binding side. This unusual binding mode may enable competitive sequestration of TPX2 even in the presence of many free NLS-containing proteins and may ensure proper spatial regulation of TPX2 in response to RanGTP [69], Figure 4. Beyond TPX2 and NuMA, numerous proteins have been identified in *Xenopus* egg extracts, which are under the control of RanGTP and importins, including the microtubule associated proteins HURP (hepatoma upregulated protein) and NuSAP (nucleolar spindle associated protein), the chromatin remodelling proteins ISWI and KANSL1/3, the RNA binding protein Rae1 and the nuclear pore complex (NPC) component Mel-28/ELYS. Consistently, all these proteins remain nuclear in interphase but perform key functions in M-phase in microtubule nucleation, microtubule organisation, spindle pole formation and anaphase spindle stability [70,71,72,73].

## 4. The Mechanism of Centrosome-Independent Microtubule Nucleation

Most mechanistic insights of spindle self-assembly once more came from *Xenopus* egg extracts due to the biochemical accessibility of the system. In egg extracts, new microtubules are generated by the activity of RanGTP regulating TPX2 and communicating with Mel-28 and γ-TuRC. TPX2, released from importins via RanGTP, associates with and activates Aurora A kinase [74], a mechanism that also stimulates Aurora A in intact human somatic cells [75]. Biochemical and structural analysis showed that the very N-terminus of TPX2 drives a conformational change in the activation segment of the kinase, which makes the t-loop phosphorylation less accessible for dephosphorylation and kinase inactivation [76,77,78]. Aurora A, in turn, modifies microtubule-associated and free centrosomal proteins to modulate their functions in spindle pole organisation. These include TPX2 itself, Kinesin 5/Eg5, TACC (transformed acidic coiled-coil protein) and the NEDD (Neddylation) 1 protein [79,80]. Aurora A-mediated phosphorylation of NEDD1 on S405, as well as recruitment of the microtubule-associated proteins X-RHAMM (*Xenopus* receptor for hyaluron-mediated mobility) and TPX2 help to activate the γ-TuRC in *Xenopus* egg extracts [81]. Of note, TPX2 also nucleates microtubule directly and differently than the γ-TuRC by promoting longitudinal and lateral interactions between tubulin dimers. This stabilises the formation of microtubule seeds as the rate limiting step in microtubule assembly [82]. Initially assembled microtubules generate further polymers: the Augmin complex, recently reconstituted from 8 subunits in vitro [83], enables microtubule nucleation on preexisting microtubules. It recruits γ-TuRC to their lattice, where TPX2 is required to assemble in a stable way the new microtubules branching out from the initial polymers [84,85]. Augmin can, thus, catalyze additional nucleation of microtubules by γ-TuRC recruitment and activation on a single microtubule filament [86]. These molecular insights explain the previously observed non-linear increase in RanGTP-induced microtubule formation [87]. They also show how the main nucleating activities of TPX2 and γ-TuRC converge in the assembly of additional microtubules from pre-existing polymers. In *Xenopus* egg extracts, immunodepletion of Augmin leads to diminished microtubule density and spindle pole fragmentation [88], similar to what has been observed upon partial depletion of the γ-TuRC from egg extracts [89]. In turn, the addition of recombinant Augmin initiates free microtubule assembly, which is further stimulated by RanGTP [86]. Recently, γ-TuRC-activation domains have been proposed to reside in the C-terminal part of *Xenopus* TPX2 [90]. These activation domains, commonly called γ-TuNA (γ-TuRC nucleation activator), are only visible for γ-TuRC once TPX2 has been released from importins, providing the most direct link between RanGTP and γ-TuRC as the main activity for templating new microtubules.

Additional experiments in human, mouse and fly oocytes showed that reducing RanGTP levels does not completely inhibit but delays or impairs meiotic spindle formation [91,92,93]. Although the remaining spindle assembly activity in these oocytes may have come from residual RanGTP, the observations are in line with the idea of additional pathways for centrosome-free microtubule formation. Experiments in *Xenopus* cell-free extracts [94,95,96] and fly oocytes [97,98] have, for instance, demonstrated that the chromatin-bound chromosomal passenger complex (CPC) contributes to microtubule nucleation from chromosomes in meiotic spindle formation in a RanGTP-independent fashion.

## 5. The Mechanism of Spindle Organization without Centrosomes

Spindle bipolarity requires balanced and robust microtubule nucleation at and around the two spindle poles. The early phase of spindle formation without centrosomes involves undirected microtubule nucleation next to chromatin. Newly formed microtubules will be stabilised by additional Ran targets including Rae1 [99] and shortly sorted and focused at their common minus ends by the minus-end directed motor proteins Dynein—with the help of NuMA—and the inverted Kinesin 14 [100,101]. The organisation and bundling of microtubule minus ends, which leads to pole formation, leaves free plus ends growing towards the chromatin. Once these plus ends get into contact with plus ends from a second microtubule array, they will recruit motor proteins and other microtubule-associated proteins specifically recognising the antiparallel order of these microtubules. In particular, the tetrameric kinesin Kinesin-5/Eg5 can sort anti-parallel polymers to establish a stable bipolar structure. Its inhibition leads to failure in spindle bipolarisation in cell-free egg extracts and in living mouse oocytes [101,102,103,104]. In egg extracts, Eg5 is directly activated by RanGTP in the vicinity of chromatin and indirectly via the activation of HURP, which helps Eg5 to function in anti-parallel sorting [62,105,106]. Female mouse *Hurp* k.o. animals are viable but sterile as their oocytes fail to establish a robust central spindle [107]. Preformed, centrosome-free MTOCs in mouse oocytes require Eg5 to get fragmented upon meiotic spindle formation. This fragmentation has to precede sorting along existing microtubules and is a prerequisite for achieving bipolarity [104,108,109]. At least in cell-free *Xenopus* egg extracts, Ran then works beyond spindle assembly. It activates ISWI, which stabilises microtubules only after the metaphase-to-anaphase-transition to allow proper chromosome segregation [110]. ISWI helps microtubules to remain stable during anaphase [70], while two specific subunits of the chromatin remodelling complex NSL (non specific lethal), KANSL1 and 3, dissociate from chromatin already before metaphase-to-anaphase-transition to stabilise microtubule minus ends [73].

The concept of RanGTP acting on importins and exportins in M-phase has been further validated in *C.elegans* and Drosophila embryos as well as in plant cells [111,112,113,114]. In all systems, the prevailing model is based on the chromatin-binding of Ran’s exchange factor RCC1 in M-phase. After nuclear envelope breakdown (NEB), RanGTP freely diffuses within the mitotic cytoplasm along with the hydrolysis promoting activities, RanBP1/2 and RanGAP. Localised RanGTP production on chromatin together with delocalised Ran’s GTP hydrolysis in the mitotic cytoplasm result in a soluble gradient of RanGTP with the highest concentration next to chromatin. This explains how Ran downstream activities involved in spindle formation in M-phase become activated only in the vicinity of chromatin [70,115,116], Figure 4. Experiments in *Xenopus* egg extracts documented how this gradient could be further sharpened: Cytosolic RanBP1, phosphorylated in M-phase on a single Serine residue (S60), forms a complex with Ran and RCC1, which inhibits the cytosolic activity of the latter and restrains cytosolic RanGTP production [117].

## 6. Communication between Chromatin and Newly Formed Centrosomes

Fast kinetics and robustness of spindle formation require the cooperation between the preformed MTOCs and the chromatin-associated activities on microtubule nucleation and organisation. Re-established MTOCs quickly assemble new microtubules. They dominate over chromatin-induced microtubule formation in early development [38] and take the lead later in differentiated somatic cells. However, the activity of chromatin remains essential [38]. In *Xenopus* egg extracts, the immunodepletion of the Ran target TPX2 completely abolishes chromatin-induced microtubule assembly [65]. Although its depletion does not inhibit basic microtubule nucleation from isolated centrosomes, it strongly impairs bipolar spindle formation driven by sperm nuclei, which comprise chromatin as well as centrosomes [57]. Consistently, RanGTP, produced from chromatin, stimulates centrosomal microtubule nucleation (Figure 4). Further biochemical experiments have enabled the identification and characterisation of Cdk11 as the key effector in Ran-regulated increase in microtubule nucleation from centrosomes [118]. Additional evidence from cell-free extracts and somatic cells underlines that the centrosome with its associated functions “sees” the activity of RanGTP in the mitotic cytoplasm after NEB. The increase in centrosomal nucleation observed at mitotic onset (“centrosome maturation”) accelerates right at NEB when RanGTP is released into the mitotic cytoplasm [119]. Centrosomal maturation involves the recruitment of additional PCM components [9] and restructuring centriolar satellites [21,22,23], which converge to increase mitotic microtubule nucleation and organisation together with centrosomal functions in signalling and cell cycle control [120]. This goes along with the release of numerous proteins from chromosomes. These turn from a function on chromatin to a Ran-regulated function on microtubules in the mitotic cytoplasm [121].

The interplay between NEB and spindle formation goes beyond rising activities of RanGTP in the mitotic cytoplasm. The NPC, consisting of some 30 protein components in copy numbers of 8 to 64 [122] dissociates into defined subcomplexes at mitotic onset concomitant with NEB [123]. The NPC protein Mel-28/ELYS was initially characterised as a pioneering component for nuclear envelope reassembly after mitosis. Both in human cells and in *C.elegans* embryos, Mel-28 rebinds to chromatin in late mitosis to seed an assembly platform for post-mitotic NPC formation [123]. In mitosis, however, Mel-28 serves additional functions that depend on the disassembly of the NPC. Released from the NPC, distinct populations of Mel-28 stay in the Nup107 subcomplex but also find new interaction partners including the γ-TuRC [124]. While the Nup107 subcomplex together with Mel-28 binds to and stabilises microtubule-kinetochore interactions, Mel-28 released from the subcomplex becomes essential for γ-TuRC-mediated microtubule nucleation. Consistently, its depletion from egg extracts abolishes spindle formation both in the absence and in the presence of centrosomes [124,125]. Thus, NPC disassembly is not merely a necessary evil of open mitosis but it is a necessary prerequisite for spindle formation.

## 7. Self-Assembly of Centrioles in Oocytes

Meiotic spindle self-assembly initiated by Ran-GTP and possibly other chromatin-localised activities highlights an intriguing feature of female reproductive cells: the ovum cytoplasm accumulates moieties required for spindle formation to an extent and to a concentration that allows, once the right trigger is provided, assembly of a functional bipolar spindle without preformed MTOCs.

Similarly, the egg cytoplasm readily self-assembles centrioles de novo even in the absence of basal bodies or basal body remnants. Although most metazoan organisms arise from sexual reproduction, a variety of organisms, in particular within the class of insects, can develop upon parthenogenesis. As oocytes of these species lose their centrioles before or during female meiosis, their female egg cytoplasm has to assemble centrosomes, including centrioles, de novo. De novo centrosome formation can also be recapitulated in *Xenopus* egg extracts. As in somatic cells, *Xenopus* Plk4 (XPlk4) initiates centriole assembly, and centrioles are readily detected by EM after the addition or overexpression of XPlk4 to egg extracts [126]. Sea-urchin eggs or egg extracts, forced to start parthenogenesis, produce huge amounts of centrioles de novo [127,128]. The egg cytoplasm holds the capacity to assemble the stereotypic centriole structure as building blocks of centrioles accumulate in eggs before fertilisation (Figure 2). This suggests that unfertilised eggs maintain a signal to inhibit premature centriole formation prior to fertilisation. Although oocytes initially block centriole de novo assembly in meiosis, the proteins and protein complexes of the PCM form centriole-free spindle poles by self-assembly in meiosis. Mouse zygotes follow this strategy even after fertilisation: two spindle poles reform from a number of centriole-free MTOCs during the first cell divisions. Acentriolar spindle formation during mouse meiosis and the early mitotic divisions require the help of the actin cytoskeleton to ensure robust spindle formation and chromosome segregation [129,130,131,132,133]. Of note, the centrosome-free pathway uses microtubule assembly promoting factors, which will later become true centrosome components. Centrosomal proteins Cep152, Cep192 and Pericentrin act in concert with Plk4 to assemble centriole-free spindle poles in meiosis and in early mitoses in mice [134,135,136,137,138]. Centrosomal as well as non-centrosomal spindle formation pathways commonly use the activity of γ-TuRC, which carries essential functions in microtubule nucleation during spindle formation irrespective of the absence or presence of centrosomes [7]. These examples underline that the two pathways share many proteins and protein modules with functions in microtubule nucleation and organisation.

## 8. Concluding Remarks

Loss and rebirth of the animal MTOC, the centrosome, accompanies meiosis and fertilisation in metazoan organisms. In most metazoans, centrosome elimination precedes meiosis, which necessitates a pathway for meiotic spindle formation in the absence of centrosomes. The mechanisms of spindle self-assembly in meiosis have evolved using mostly the same molecular determinants that are found to nucleate and organise microtubules in the PCM of centrosomes. Centrosome-free microtubule assembly requires RanGTP produced around chromatin, the nucleation promoting activity of kinetochores, as well as positive feedback loops of microtubule nucleation from pre-existing polymers. Analysing spindle assembly in the absence of centrosomes has not only helped to comprehend the biological challenges of meiosis, fertilisation and fertility, it has also highlighted the idea of a cooperation of different microtubule assembly pathways, both centrosomal and non-centrosomal, for spindle assembly in all our somatic cells.

## References

[1] Severson A.F., von Dassow G., Bowerman B. (2016). Oocyte Meiotic Spindle Assembly and Function. Curr. Top. Dev. Biol..

[2] Schatten G. (1994). The centrosome and its mode of inheritance: The reduction of the centrosome during Gametogenesis and its restoration during fertilization. Dev. Biol..

[3] Clift D., Schuh M. (2013). Restarting life: Fertilization and the transition from meiosis to mitosis. Nat. Rev. Mol. Cell Biol..

[4] Loncarek J., Bettencourt-Dias M. (2018). Building the right centriole for each cell type. J. Cell Biol..

[5] Luders J. (2012). The amorphous pericentriolar cloud takes shape. Nat. Cell Biol..

[6] Kollman J.M., Merdes A., Mourey L., Agard D.A. (2011). Microtubule nucleation by gamma-tubulin complexes. Nat. Rev. Mol. Cell Biol..

[7] Lin T.C., Neuner A., Schiebel E. (2015). Targeting of gamma-tubulin complexes to microtubule organizing centers: Conservation and divergence. Trends Cell Biol..

[8] Woodruff J.B., Wueseke O., Hyman A.A. (2014). Pericentriolar material structure and dynamics. Philos. Trans. R. Soc. Lond. Ser. B Biol. Sci..

[9] Fry A.M., Sampson J., Shak C., Shackleton S. (2017). Recent advances in pericentriolar material organization: Ordered layers and scaffolding gels. F1000Research.

[10] Woodruff J.B., Ferreira Gomes B., Widlund P.O., Mahamid J., Honigmann A., Hyman A.A. (2017). The Centrosome Is a Selective Condensate that Nucleates Microtubules by Concentrating Tubulin. Cell.

[11] Petry S. (2016). Mechanisms of Mitotic Spindle Assembly. Annu. Rev. Biochem..

[12] Prosser S.L., Pelletier L. (2017). Mitotic spindle assembly in animal cells: A fine balancing act. Nat. Rev. Mol. Cell Biol..

[13] McIntosh J.R. (2016). Mitosis. Cold Spring Harbor Perspect. Biol..

[14] Nigg E.A., Cajanek L., Arquint C. (2014). The centrosome duplication cycle in health and disease. FEBS Lett..

[15] Sluder G. (2014). One to only two: A short history of the centrosome and its duplication. Philos. Trans. R. Soc. Lond. Ser. B Biol. Sci..

[16] Firat-Karalar E.N., Stearns T. (2014). The centriole duplication cycle. Philos. Trans. R. Soc. Lond. Ser. B Biol. Sci..

[17] Hinchcliffe E.H. (2014). Centrosomes and the art of mitotic spindle maintenance. Int. Rev. Cell Mol. Biol..

[18] Fu J., Hagan I.M., Glover D.M. (2015). The centrosome and its duplication cycle. Cold Spring Harbor Perspect. Biol..

[19] Paz J., Luders J. (2018). Microtubule-Organizing Centers: Towards a Minimal Parts List. Trends Cell Biol..

[20] Nigg E.A., Holland A.J. (2018). Once and only once: Mechanisms of centriole duplication and their deregulation in disease. Nat. Rev. Mol. Cell Biol..

[21] Bärenz F., Mayilo D., Gruss O.J. (2011). Centriolar Satellites: Busy Orbits around the Centrosome. Eur. J. Cell Biol..

[22] Tollenaere M.A., Mailand N., Bekker-Jensen S. (2015). Centriolar satellites: Key mediators of centrosome functions. Cell. Mol. Life Sci..

[23] Hori A., Toda T. (2017). Regulation of centriolar satellite integrity and its physiology. Cell. Mol. Life Sci..

[24] Szollosi D., Calarco P., Donahue R.P. (1972). Absence of centrioles in the first and second meiotic spindles of mouse oocytes. J. Cell Sci..

[25] Sluder G., Miller F.J., Lewis K., Davison E.D., Rieder C.L. (1989). Centrosome inheritance in starfish zygotes: Selective loss of the maternal centrosome after fertilization. Dev. Biol..

[26] Nakashima S., Kato K.H. (2001). Centriole behavior during meiosis in oocytes of the sea urchin Hemicentrotus pulcherrimus. Dev. Growth Differ..

[27] Shirato Y., Tamura M., Yoneda M., Nemoto S. (2006). Centrosome destined to decay in starfish oocytes. Development.

[28] Sathananthan A.H., Selvaraj K., Girijashankar M.L., Ganesh V., Selvaraj P., Trounson A.O. (2006). From oogonia to mature oocytes: Inactivation of the maternal centrosome in humans. Microsc. Res. Tech..

[29] Pimenta-Marques A., Bento I., Lopes C.A., Duarte P., Jana S.C., Bettencourt-Dias M. (2016). A mechanism for the elimination of the female gamete centrosome in Drosophila melanogaster. Science.

[30] Washitani-Nemoto S., Saitoh C., Nemoto S. (1994). Artificial parthenogenesis in starfish eggs: Behavior of nuclei and chromosomes resulting in tetraploidy of parthenogenotes produced by the suppression of polar body extrusion. Dev. Biol..

[31] Manandhar G., Schatten H., Sutovsky P. (2005). Centrosome reduction during gametogenesis and its significance. Biol. Reprod..

[32] Kim D.Y., Roy R. (2006). Cell cycle regulators control centrosome elimination during oogenesis in Caenorhabditis elegans. J. Cell Biol..

[33] Borrego-Pinto J., Somogyi K., Karreman M.A., Konig J., Muller-Reichert T., Bettencourt-Dias M., Gonczy P., Schwab Y., Lenart P. (2016). Distinct mechanisms eliminate mother and daughter centrioles in meiosis of starfish oocytes. J. Cell Biol..

[34] Schoborg T.A., Rusan N.M. (2016). Taking Centrioles to the Elimination Round. Dev. Cell.

[35] Moorhouse K.S., Burgess D.R. (2014). How to be at the right place at the right time: The importance of spindle positioning in embryos. Mol. Reprod. Dev..

[36] Chaigne A., Terret M.E., Verlhac M.H. (2017). Asymmetries and Symmetries in the Mouse Oocyte and Zygote. Results Probl. Cell Differ..

[37] Hasley A., Chavez S., Danilchik M., Wuhr M., Pelegri F. (2017). Vertebrate Embryonic Cleavage Pattern Determination. Adv. Exp. Med. Biol..

[38] Cavazza T., Peset I., Vernos I. (2016). From meiosis to mitosis—The sperm centrosome defines the kinetics of spindle assembly after fertilization in Xenopus. J. Cell Sci..

[39] Inoue D., Wittbrodt J., Gruss O.J. (2018). Loss and Rebirth of the Animal Microtubule Organizing Center: How Maternal Expression of Centrosomal Proteins Cooperates with the Sperm Centriole in Zygotic Centrosome Reformation. Bioessays.

[40] Avidor-Reiss T., Gopalakrishnan J. (2013). Building a centriole. Curr. Opin. Cell Biol..

[41] Verlhac M.H., Terret M.E. (2016). Oocyte Maturation and Development. F1000Research.

[42] Hunt P.A. (2006). Meiosis in mammals: Recombination, non-disjunction and the environment. Biochem. Soc. Trans..

[43] Pacchierotti F., Adler I.D., Eichenlaub-Ritter U., Mailhes J.B. (2007). Gender effects on the incidence of aneuploidy in mammalian germ cells. Environ. Res..

[44] Webster A., Schuh M. (2017). Mechanisms of Aneuploidy in Human Eggs. Trends Cell Biol..

[45] Greaney J., Wei Z., Homer H. (2017). Regulation of chromosome segregation in oocytes and the cellular basis for female meiotic errors. Hum. Reprod. Update.

[46] Karsenti E., Newport J., Hubble R., Kirschner M. (1984). Interconversion of metaphase and interphase microtubule arrays, as studied by the injection of centrosomes and nuclei into Xenopus eggs. J. Cell Biol..

[47] Karsenti E., Newport J., Kirschner M. (1984). The respective roles of centrosomes and chromatin in the conversion of microtubule arrays from interphase to metaphase. J. Cell Biol..

[48] Karsenti E., Vernos I. (2001). The mitotic spindle: A self-made machine. Science.

[49] Hallen M.A., Endow S.A. (2009). Anastral spindle assembly: A mathematical model. Biophys. J..

[50] Masui Y., Markert C.L. (1971). Cytoplasmic control of nuclear behavior during meiotic maturation of frog oocytes. J. Exp. Zool..

[51] Murray A., Kay B.K., Peng H.B. (1991). Xenopus Laevis: Practical Uses in Cell and Molecular Biology.

[52] Sawin K.E., Mitchison T.J. (1991). Mitotic spindle assembly by two different pathways in vitro. J. Cell Biol..

[53] Heald R., Tournebize R., Blank T., Sandaltzopoulos R., Becker P., Hyman A., Karsenti E. (1996). Self-organization of microtubules into bipolar spindles around artificial chromosomes in Xenopus egg extracts. Nature.

[54] Heald R., Tournebize R., Habermann A., Karsenti E., Hyman A. (1997). Spindle assembly in Xenopus egg extracts: Respective roles of centrosomes and microtubule self-organization. J. Cell Biol..

[55] Cavazza T., Vernos I. (2015). The RanGTP Pathway: From Nucleo-Cytoplasmic Transport to Spindle Assembly and Beyond. Front. Cell Dev. Biol..

[56] Carazo-Salas R.E., Guarguaglini G., Gruss O.J., Segref A., Karsenti E., Mattaj I.W. (1999). Generation of GTP-bound Ran by RCC1 is required for chromatin-induced mitotic spindle formation. Nature.

[57] Gruss O.J., Wittmann M., Yokoyama H., Pepperkok R., Kufer T., Silljé H., Karsenti E., Mattaj I.W., Vernos I. (2002). Chromosome-induced microtubule assembly mediated by TPX2 is required for spindle formation in HeLa cells. Nat. Cell Biol..

[58] Wilde A., Zheng Y. (1999). Stimulation of microtubule aster formation and spindle assembly by the small GTPase Ran. Science.

[59] Zhang Y., Heidebrecht H., Rott A., Schlegelberger B., Parwaresch R. (1999). Assignment of human proliferation associated p100 gene (C20orf1) to human chromosome band 20q11.2 by in situ hybridization. Cytogenet. Cell Genet..

[60] Kalab P., Pu R.T., Dasso M. (1999). The ran GTPase regulates mitotic spindle assembly. Curr. Biol..

[61] Carazo-Salas R.E., Gruss O.J., Mattaj I.W., Karsenti E. (2001). Ran-GTP coordinates regulation of microtubule nucleation and dynamics during mitotic-spindle assembly. Nat. Cell Biol..

[62] Wilde A., Lizarraga S.B., Zhang L., Wiese C., Gliksman N.R., Walczak C.E., Zheng Y. (2001). Ran stimulates spindle assembly by altering microtubule dynamics and the balance of motor activities. Nat. Cell Biol..

[63] Ohba T., Nakamura M., Nishitani H., Nishimoto T. (1999). Self-organization of microtubule asters induced in Xenopus egg extracts by GTP-bound Ran. Science.

[64] Zhang C., Hughes M., Clarke P.R. (1999). Ran-GTP stabilises microtubule asters and inhibits nuclear assembly in Xenopus egg extracts. J. Cell Sci..

[65] Gruss O.J., Carazo-Salas R.E., Schatz C.A., Guarguaglini G., Kast J., Wilm M., Le Bot N., Vernos I., Karsenti E., Mattaj I.W. (2001). Ran Induces Spindle Assembly by Reversing the Inhibitory Effect of Importin alpha on TPX2 Activity. Cell.

[66] Nachury M.V., Maresca T.J., Salmon W.C., Waterman-Storer C.M., Heald R., Weis K. (2001). Importin beta Is a Mitotic Target of the Small GTPase Ran in Spindle Assembly. Cell.

[67] Wiese C., Wilde A., Moore M.S., Adam S.A., Merdes A., Zheng Y. (2001). Role of Importin b in Coupling Ran to Downstream Targets in Microtubule Assembly. Science.

[68] Hetzer M., Gruss O.J., Mattaj I.W. (2002). The Ran GTPase as a marker for chromosome position in spindle formation and nuclear envelope assembly. Nat. Cell Biol..

[69] Giesecke A., Stewart M. (2010). Novel binding of the mitotic regulator TPX2 (target protein for Xenopus kinesin-like protein 2) to importin-alpha. J. Biol. Chem..

[70] Gruss O.J., Wittinghofer A. (2014). Ras Superfamily Small G Proteins: Biology and Mechanisms.

[71] Okada N., Sato M. (2015). Spatiotemporal Regulation of Nuclear Transport Machinery and Microtubule Organization. Cells.

[72] Forbes D.J., Travesa A., Nord M.S., Bernis C. (2015). Nuclear transport factors: Global regulation of mitosis. Curr. Opin. Cell Biol..

[73] Meunier S., Shvedunova M., Van Nguyen N., Avila L., Vernos I., Akhtar A. (2015). An epigenetic regulator emerges as microtubule minus-end binding and stabilizing factor in mitosis. Nat. Commun..

[74] Tsai M.Y., Wiese C., Cao K., Martin O., Donovan P., Ruderman J., Pringent C., Zheng Y. (2003). A Ran signalling pathway mediated by the mitotic kinase Aurora A in spindle assembly. Nat. Cell Biol..

[75] Kufer T.A., Sillje H.H., Korner R., Gruss O.J., Meraldi P., Nigg E.A. (2002). Human TPX2 is required for targeting Aurora-A kinase to the spindle. J. Cell Biol..

[76] Eyers P.A., Erikson E., Chen L.G., Maller J.L. (2003). A novel mechanism for activation of the protein kinase Aurora A. Curr. Biol..

[77] Bayliss R., Sardon T., Vernos I., Conti E. (2003). Structural Basis of Aurora-A Activation by TPX2 at the Mitotic Spindle. Mol. Cell.

[78] Bayliss R., Sardon T., Ebert J., Lindner D., Vernos I., Conti E. (2004). Determinants for Aurora-A activation and Aurora-B discrimination by TPX2. Cell Cycle.

[79] Eyers P.A., Maller J.L. (2004). Regulation of Xenopus Aurora A activation by TPX2. J. Biol. Chem..

[80] Barr A.R., Gergely F. (2007). Aurora-A: The maker and breaker of spindle poles. J. Cell Sci..

[81] Scrofani J., Sardon T., Meunier S., Vernos I. (2015). Microtubule nucleation in mitosis by a RanGTP-dependent protein complex. Curr. Biol..

[82] Zhang R., Roostalu J., Surrey T., Nogales E. (2017). Structural insight into TPX2-stimulated microtubule assembly. eLife.

[83] Hsia K.C., Wilson-Kubalek E.M., Dottore A., Hao Q., Tsai K.L., Forth S., Shimamoto Y., Milligan R.A., Kapoor T.M. (2014). Reconstitution of the augmin complex provides insights into its architecture and function. Nat. Cell Biol..

[84] Petry S., Groen A.C., Ishihara K., Mitchison T.J., Vale R.D. (2013). Branching microtubule nucleation in Xenopus egg extracts mediated by augmin and TPX2. Cell.

[85] Farache D., Emorine L., Haren L., Merdes A. (2018). Assembly and regulation of gamma-tubulin complexes. Open Biol..

[86] Sanchez-Huertas C., Luders J. (2015). The augmin connection in the geometry of microtubule networks. Curr. Biol..

[87] Clausen T., Ribbeck K. (2007). Self-organization of anastral spindles by synergy of dynamic instability, autocatalytic microtubule production, and a spatial signaling gradient. PLoS ONE.

[88] Petry S., Pugieux C., Nedelec F.J., Vale R.D. (2011). Augmin promotes meiotic spindle formation and bipolarity in Xenopus egg extracts. Proc. Natl. Acad. Sci. USA.

[89] Bärenz F., Inoue D., Yokoyama H., Tegha-Dunghu J., Freiss S., Draeger S., Mayilo D., Cado I., Merker S., Klinger M. (2013). The centriolar satellite protein SSX2IP promotes centrosome maturation. J. Cell Biol..

[90] Alfaro-Aco R., Thawani A., Petry S. (2017). Structural analysis of the role of TPX2 in branching microtubule nucleation. J. Cell Biol..

[91] Dumont J., Petri S., Pellegrin F., Terret M.E., Bohnsack M.T., Rassinier P., Georget V., Kalab P., Gruss O.J., Verlhac M.H. (2007). A centriole- and RanGTP-independent spindle assembly pathway in meiosis I of vertebrate oocytes. J. Cell Biol..

[92] Cesario J., McKim K.S. (2011). RanGTP is required for meiotic spindle organization and the initiation of embryonic development in Drosophila. J. Cell Sci..

[93] Holubcova Z., Blayney M., Elder K., Schuh M. (2015). Human oocytes. Error-prone chromosome-mediated spindle assembly favors chromosome segregation defects in human oocytes. Science.

[94] Sampath S.C., Ohi R., Leismann O., Salic A., Pozniakovski A., Funabiki H. (2004). The chromosomal passenger complex is required for chromatin-induced microtubule stabilization and spindle assembly. Cell.

[95] Kelly A.E., Sampath S.C., Maniar T.A., Woo E.M., Chait B.T., Funabiki H. (2007). Chromosomal enrichment and activation of the aurora B pathway are coupled to spatially regulate spindle assembly. Dev. Cell.

[96] Tseng B.S., Tan L., Kapoor T.M., Funabiki H. (2010). Dual detection of chromosomes and microtubules by the chromosomal passenger complex drives spindle assembly. Dev. Cell.

[97] Radford S.J., Jang J.K., McKim K.S. (2012). The chromosomal passenger complex is required for meiotic acentrosomal spindle assembly and chromosome biorientation. Genetics.

[98] Das A., Shah S.J., Fan B., Paik D., DiSanto D.J., Hinman A.M., Cesario J.M., Battaglia R.A., Demos N., McKim K.S. (2016). Spindle Assembly and Chromosome Segregation Requires Central Spindle Proteins in Drosophila Oocytes. Genetics.

[99] Blower M.D., Nachury M., Heald R., Weis K. (2005). A Rae1-containing ribonucleoprotein complex is required for mitotic spindle assembly. Cell.

[100] Merdes A., Cleveland D.W. (1997). Pathways of spindle pole formation: Different mechanisms; conserved components. J. Cell Biol..

[101] Walczak C.E., Vernos I., Mitchison T.J., Karsenti E., Heald R. (1998). A model for the proposed roles of different microtubule-based motor proteins in establishing spindle bipolarity. Curr. Biol..

[102] Kapoor T.M., Mayer T.U., Coughlin M.L., Mitchison T.J. (2000). Probing spindle assembly mechanisms with monastrol, a small molecule inhibitor of the mitotic kinesin, Eg5. J. Cell Biol..

[103] Mailhes J.B., Mastromatteo C., Fuseler J.W. (2004). Transient exposure to the Eg5 kinesin inhibitor monastrol leads to syntelic orientation of chromosomes and aneuploidy in mouse oocytes. Mutat Res..

[104] Schuh M., Ellenberg J. (2007). Self-organization of MTOCs replaces centrosome function during acentrosomal spindle assembly in live mouse oocytes. Cell.

[105] Koffa M.D., Casanova C.M., Santarella R., Kocher T., Wilm M., Mattaj I.W. (2006). HURP is part of a Ran-dependent complex involved in spindle formation. Curr. Biol..

[106] Sillje H.H., Nagel S., Korner R., Nigg E.A. (2006). HURP is a Ran-importin beta-regulated protein that stabilizes kinetochore microtubules in the vicinity of chromosomes. Curr. Biol..

[107] Breuer M., Kolano A., Kwon M., Li C.C., Tsai T.F., Pellman D., Brunet S., Verlhac M.H. (2010). HURP permits MTOC sorting for robust meiotic spindle bipolarity, similar to extra centrosome clustering in cancer cells. J. Cell Biol..

[108] Luksza M., Queguigner I., Verlhac M.H., Brunet S. (2013). Rebuilding MTOCs upon centriole loss during mouse oogenesis. Dev. Biol..

[109] Clift D., Schuh M. (2015). A three-step MTOC fragmentation mechanism facilitates bipolar spindle assembly in mouse oocytes. Nat. Commun..

[110] Yokoyama H., Rybina S., Santarella-Mellwig R., Mattaj I.W., Karsenti E. (2009). ISWI is a RanGTP-dependent MAP required for chromosome segregation. J. Cell Biol..

[111] Zhang H., Dawe R.K. (2011). Mechanisms of plant spindle formation. Chromosom. Res..

[112] Chen J.W., Barker A.R., Wakefield J.G. (2015). The Ran Pathway in Drosophila melanogaster Mitosis. Front. Cell Dev. Biol..

[113] Bennabi I., Terret M.E., Verlhac M.H. (2016). Meiotic spindle assembly and chromosome segregation in oocytes. J. Cell Biol..

[114] Heald R., Gibeaux R. (2018). Subcellular scaling: Does size matter for cell division?. Curr. Opin. Cell Biol..

[115] Kalab P., Solc P., Motlik J. (2011). The role of RanGTP gradient in vertebrate oocyte maturation. Results Probl. Cell Differ..

[116] Weaver L.N., Walczak C.E. (2015). Spatial gradients controlling spindle assembly. Biochem. Soc. Trans..

[117] Zhang M.S., Arnaoutov A., Dasso M. (2014). RanBP1 governs spindle assembly by defining mitotic Ran-GTP production. Dev. Cell.

[118] Yokoyama H., Gruss O.J., Rybina S., Caudron M., Schelder M., Wilm M., Mattaj I.W., Karsenti E. (2008). Cdk11 is a RanGTP-dependent microtubule stabilization factor that regulates spindle assembly rate. J. Cell Biol..

[119] Cavazza T., Malgaretti P., Vernos I. (2016). The sequential activation of the mitotic microtubule assembly pathways favors bipolar spindle formation. Mol. Biol. Cell..

[120] Arquint C., Gabryjonczyk A.M., Nigg E.A. (2014). Centrosomes as signalling centres. Philos. Trans. R. Soc. Lond. Ser. B Biol. Sci..

[121] Yokoyama H., Gruss O.J. (2013). New mitotic regulators released from chromatin. Front. Oncol..

[122] Beck M., Hurt E. (2017). The nuclear pore complex: Understanding its function through structural insight. Nat. Rev. Mol. Cell Biol..

[123] Imamoto N., Funakoshi T. (2012). Nuclear pore dynamics during the cell cycle. Curr. Opin. Cell Biol..

[124] Franz C., Walczak R., Yavuz S., Santarella R., Gentzel M., Askjaer P., Galy V., Hetzer M., Mattaj I.W., Antonin W. (2007). MEL-28/ELYS is required for the recruitment of nucleoporins to chromatin and postmitotic nuclear pore complex assembly. EMBO Rep..

[125] Yokoyama H., Koch B., Walczak R., Ciray-Duygu F., Gonzalez-Sanchez J.C., Devos D.P., Mattaj I.W., Gruss O.J. (2014). The nucleoporin MEL-28 promotes RanGTP-dependent gamma-tubulin recruitment and microtubule nucleation in mitotic spindle formation. Nat. Commun..

[126] Eckerdt F., Yamamoto T.M., Lewellyn A.L., Maller J.L. (2011). Identification of a polo-like kinase 4-dependent pathway for de novo centriole formation. Curr. Biol..

[127] Kallenbach R., Mazia D. (1982). Origin and maturation of centrioles in association with the nuclear envelope in hypertonic stressed sea urchin eggs. Eur. J. Cell Biol..

[128] Kallenbach R.J. (1983). The induction of de novo centrioles in sea urchin eggs: A possible common mechanism for centriolar activation among parthenogenetic procedures. Eur. J. Cell Biol..

[129] Courtois A., Schuh M., Ellenberg J., Hiiragi T. (2012). The transition from meiotic to mitotic spindle assembly is gradual during early mammalian development. J. Cell Biol..

[130] Field C.M., Lenart P. (2011). Bulk cytoplasmic actin and its functions in meiosis and mitosis. Curr. Biol..

[131] Yi K., Li R. (2012). Actin cytoskeleton in cell polarity and asymmetric division during mouse oocyte maturation. Cytoskeleton (Hoboken).

[132] Li R., Albertini D.F. (2013). The road to maturation: Somatic cell interaction and self-organization of the mammalian oocyte. Nat. Rev. Mol. Cell Biol..

[133] Almonacid M., Terret M.E., Verlhac M.H. (2014). Actin-based spindle positioning: New insights from female gametes. J. Cell Sci..

[134] Carabatsos M.J., Combelles C.M., Messinger S.M., Albertini D.F. (2000). Sorting and reorganization of centrosomes during oocyte maturation in the mouse. Microsc. Res. Tech..

[135] Luo Y.B., Kim N.H. (2015). PLK4 is essential for meiotic resumption in mouse oocytes. Biol. Reprod..

[136] Bury L., Coelho P.A., Simeone A., Ferries S., Eyers C.E., Eyers P.A., Zernicka-Goetz M., Glover D.M. (2017). Plk4 and Aurora A cooperate in the initiation of acentriolar spindle assembly in mammalian oocytes. J. Cell Biol..

[137] Coelho P.A., Bury L., Sharif B., Riparbelli M.G., Fu J., Callaini G., Glover D.M., Zernicka-Goetz M. (2013). Spindle formation in the mouse embryo requires Plk4 in the absence of centrioles. Dev. Cell.

[138] Lee I.W., Jo Y.J., Jung S.M., Wang H.Y., Kim N.H., Namgoong S. (2017). Distinct roles of Cep192 and Cep152 in acentriolar MTOCs and spindle formation during mouse oocyte maturation. FASEB J..

